# Isolation of brightness induction effects on target patches from adjacent surrounds and remote backgrounds

**DOI:** 10.3389/fnhum.2022.1082059

**Published:** 2023-03-14

**Authors:** Barbara Blakeslee, Mark E. McCourt

**Affiliations:** Center for Visual and Cognitive Neuroscience, Department of Psychology, North Dakota State University, Fargo, ND, United States

**Keywords:** brightness induction, brightness contrast, brightness assimilation, brightness mechanisms, achromatic perception

## Abstract

The brightness (perceived intensity) of a region of visual space depends on its luminance and on the luminance of nearby regions. This phenomenon is called brightness induction and includes both brightness contrast and assimilation. Historically, and on a purely descriptive level, brightness contrast refers to a directional shift in target brightness away from the brightness of an adjacent region while assimilation refers to a brightness shift toward that of an adjacent region. In order to understand mechanisms, it is important to differentiate the descriptive terms contrast and assimilation from the optical and/or neural processes, often similarly named, which cause the effects. Experiment 1 isolated the effect on target patch (64 cd/m^2^) matching luminance (brightness) of six surround-ring widths (0.1°–24.5°) varied over 11 surround-ring luminances (32–96 cd/m^2^). Using the same observers, Experiment 2 examined the effect of the identical surround-ring parameters on target patch matching luminance in the presence of a dark (0.0 cd/m^2^) and a bright (96 cd/m^2^) remote background. By differencing the results of Experiment 1 (the isolated effect of the surround-ring) from those of Experiment 2 (the combined effect of the surround-ring with the dark and bright remote background) we further isolated the effect of the remote background. The results reveal that surround-rings and remote backgrounds produce brightness contrast effects in the target patch that are of the same or opposite polarity depending on the luminance polarity of these regions relative to target patch luminance. The strength of brightness contrast from the surround-ring varied with surround-ring luminance and width. Brightness contrast (darkening) in the target from the bright remote background was relatively constant in magnitude across all surround-ring luminances and increased in magnitude with decreasing surround-ring width. Brightness contrast (brightening) from the isolated dark remote background also increased in magnitude with decreasing surround-ring width: however, despite some regional flattening of the functions due to the fixed luminance of the dark remote background, induction magnitude was much reduced in the presence of a surround-ring of greater luminance than the target patch indicating a non-linear interaction between the dark remote background and surround-ring luminance.

## Introduction

The brightness (perceived intensity) of a region of visual space is not determined exclusively by that region’s luminance but depends also upon the luminance of nearby regions. This phenomenon is called brightness induction and includes both brightness contrast and brightness assimilation effects. Historically, and on a purely descriptive (as opposed to mechanistic) level, brightness contrast induction is said to occur when the brightness of a target region shifts *away* from the brightness of an adjacent region, for example, when a gray patch in a bright surround looks darker than an equally luminant gray patch in a dark surround (Heinemann, 1955, 1972). Brightness assimilation, on the other hand, is said to occur when the brightness of a target region shifts *toward* that of an adjacent region and is most often observed in displays containing high spatial frequency patterns (Helson, 1963; White, 1979; DeValois and DeValois, 1988; Smith et al., 2001; Blakeslee and McCourt, 2004). Helson (1963) studied brightness contrast and brightness assimilation from white and black lines on mid-gray backgrounds as a function of line width and line separation. He concluded that brightness assimilation and brightness contrast form a continuum including a transition zone where neither effect is observed. Specifically, Helson (1963) found that brightness contrast induction transitioned to brightness assimilation with decreasing line width and that, in general, both brightness contrast and brightness assimilation decreased as line separation increased. Other more recent studies have also observed a transition from brightness induction in the direction of brightness contrast to brightness induction in the direction of brightness assimilation as various inducing patterns varied in spatial frequency from low to high (DeValois and DeValois, 1988; Jameson and Hurvich, 1989; Blakeslee and McCourt, 2004; Rudd, 2010).

Both forms of brightness induction are of research interest because of the window they provide into the mechanisms underlying visual function. When discussing mechanisms, it is important to differentiate between the purely descriptive terms *contrast* and *assimilation* that merely specify the direction of a brightness induction effect on a region in relation to its adjacent surround, and the actual optical or neural mechanisms that produce the directional shifts. For example, optical (as opposed to neural) blurring has been shown to contribute to brightness assimilation under some high-frequency conditions (Smith et al., 2001). Optical blurring does not obviate the need for a neural explanation of the effect as well, however, since the signature of optical blurring (a linear increase in magnitude as a function of inducing luminance), is not always observed (Jameson and Hurvich, 1989; Hong and Shevell, 2004). In other instances (discussed in detail below), effects that are described as brightness assimilation have been shown to result from strong directional brightness contrast mechanisms (Blakeslee et al., 2016); while others, which are the topic of the present article, appear to be the manifestation of remote brightness contrast mechanisms (Shapley and Reid, 1985; Reid and Shapley, 1988).

Historically, a popular proposed mechanism for brightness contrast induction has its roots in the stabilized image experiments of the 1960s (Krauskopf, 1963; Gerrits et al., 1966; Yarbus, 1967). These studies demonstrated that a stabilized disk on an unstabilized background fades and is filled-in by the color and brightness of the background. Krauskopf (1963) suggested that the color of areas between contours was determined by the temporal changes (information) at the contours alone. This idea also found support in the un-stabilized image experiments of Craik (1966), O’Brien (1958), and Cornsweet (1966, 1970) using shallow spatial luminance gradients and luminance steps. Like stabilized contours, the shallow luminance gradients failed to signal a perceptual change in luminance resulting in what is now termed the Craik-O’Brien-Cornsweet illusion (see also Arend et al., 1971). The above studies led to an explanation for brightness contrast induction (and indeed brightness perception in general) in which the brightness of a target patch (or region) was thought to be determined by the luminance information at the target patch edge (for example by average perimeter contrast) and subsequently “filled-in” or assigned to the entire enclosed area (Cornsweet and Teller, 1965; Shapley and Enroth-Cugell, 1984; Grossberg and Todorovic, 1988; Paradiso and Nakayama, 1991; Paradiso and Hahn, 1996; Rossi and Paradiso, 1996; for review see Kingdom and Moulden, 1988; Grossberg, 2003).

Jameson and Hurvich (1975) and Jameson (1985) proposed an alternative mechanism that could account for both brightness contrast and brightness assimilation based on parallel processing at multiple spatial scales by difference-of-Gaussians (DOG) filters. According to this explanation, the center-surround antagonism of the DOG filters [which resemble retinal ganglion and lateral geniculate nucleus (LGN) receptive fields] produces a brightness contrast effect when, for example, the center size of the filter matches the size of the target patch. The same filter, however, produces an assimilation effect (neural blurring) when the target patch is small because the filter center averages the light falling within its boundaries. Jameson and Hurvich (1989) emphasized that due to the multiple spatial scales of these filters, contrast and assimilation can occur simultaneously in filters that differ in size.

Although “filling-in” as a mechanism may be a viable explanation for low-contrast induction effects such as the Craik-Cornsweet-O’Brien illusion (Burr, 1987; but see Dakin and Bex, 2003) it is clear that many of the original ideas underpinning arguments for this mechanism as universal have been eroded by subsequent data (for review see Kingdom, 2011) which as a whole lend support to explanations based on spatial filtering. For example, the nearly instantaneous timing (Blakeslee and McCourt, 2008) and spatial structure of induction effects such as grating induction (McCourt, 1982; Foley and McCourt, 1985), in which a sinewave inducing grating produces an induced counterphase sinewave grating (brightness modulation) in a homogeneous elongated target field perpendicular to the inducing grating, cannot be explained by a simple homogeneous “filling-in” of brightness from the edges of the target field. Point-by-point brightness matching in the targets of stimuli that transition from the elongated target fields characteristic of grating induction to the separate target patches characteristic of simultaneous brightness contrast, demonstrates that these stimuli form a continuum, and that brightness induction is structured (not homogeneous) in both grating induction and simultaneous brightness contrast stimuli (Blakeslee and McCourt, 1997). Strengthening Jameson and Hurvich’s original hypothesis (Hurvich and Jameson, 1966; Jameson and Hurvich, 1975, 1989; Jameson, 1985) that both brightness contrast and brightness assimilation are based on parallel processing at multiple spatial scales by difference-of-Gaussians (DOG) filters, Blakeslee and McCourt (1997) demonstrated that the patterned induction in the target patches of these stimuli could be modeled within the context of a quantitative multiscale spatial filtering model, which they named the difference-of-Gaussians (DOG) model.

Subsequently, Blakeslee and McCourt (1999) investigated the White effect (White, 1979, 1981), in which elongated target patches of identical luminance placed on the black and white bars of a square wave grating appear different in brightness. This effect has been widely studied since unlike simultaneous brightness contrast or grating induction, the direction of brightness induction in the White effect is not correlated with the aspect ratio of the target patch. Instead, the target patch appears to contrast with the bars on which it is situated (collinear) largely independent of the amount of contact it has with the black or white flanking bars. Similar to simultaneous brightness contrast and grating induction, however, Blakeslee and McCourt (1999) demonstrated, again using point-by-point brightness matching, that induction in the target patches of White stimuli is also structured. These behaviors of the White effect render a simple brightness contrast, brightness assimilation, or “filling-in” mechanism, as well as the original DOG model unable to account for the effect. To predict the White effect, in addition to simultaneous brightness contrast and grating induction, Blakeslee and McCourt (1999) found they needed to modify the multiscale DOG model by using multiscale oriented DOG (ODOG) filters. In this model the outputs of these oriented multiscale filters are normalized and summed across orientation. A later study using White, shifted White, and checkerboard stimuli (Blakeslee and McCourt, 2004) was able to show that the ODOG model could also account for the transition from brightness contrast to assimilation in shifted White and checkerboard stimuli and for the increase in the magnitude of the original White effect at high frequencies ascribed to brightness assimilation.

The oriented filter explanation for the White effect is further supported by a more recent study (Blakeslee et al., 2016) in which the luminance of the collinear and flanking bars was independently manipulated in order to investigate their separate influence on target patch matching luminance (apparent intensity or brightness). The inducing grating was a 0.5 c/d square-wave and target patches measured 1.0° in width and either 0.5° or 3.0° in height. Target patches measuring 0.5° in height had more extensive contact with the collinear bars and target patches measuring 3.0° in height had more extensive contact with the flanking bars. The luminance of the collinear (or flanking) bars assumed 20 values from 3.0 to 125 cd/m^2^, while the luminance of the flanking (or collinear) bars remained white (125 cd/m^2^) or black (3.0 cd/m^2^). Under these conditions the influence of the collinear and flanking bars was found to be purely in the direction of contrast as predicted by the ODOG model. The effect was dominated by contrast from the collinear bars (which results in White’s effect); however, the influence of the flanking bars was also in the contrast direction. These findings ruled out mechanistic explanations of the White effect in terms of brightness assimilation from the flanking bars (Otazu et al., 2008; Domijan, 2015). As shown previously (Blakeslee and McCourt, 2004), however, assimilation from the flanking bars does augment rather than reduce the magnitude of the White effect when the spatial frequency of the inducing square-wave grating exceeds 2.0 c/d.

Another major challenge to the idea that simple edge-based “filling-in” underlies brightness contrast induction are the findings that remote regions, and not only regions immediately adjacent to a target region, can influence the apparent intensity (brightness) of a target (Arend et al., 1971; Land and McCann, 1971; Shapley and Reid, 1985; Reid and Shapley, 1988; Zaidi et al., 1992; Heinemann and Chase, 1995; Logvinenko, 2003; Hong and Shevell, 2004; Rudd, 2010). Zaidi et al. (1992) studied induction in a homogeneous target disk (1° diameter) using circularly symmetric spatial sinewaves as inducers (0.05–3.2 c/d) within an inducing surround (8° diameter). Their results showed that brightness contrast induction was an additive (linear) spatial process. Induction in the target disk was predicted by summing regions of the inducing surround after weighting by a negative exponential as a function of distance from the target. In addition, contrast induction from the combination of two different frequency sinewaves was equal to the summation of the induction from each sinewave presented separately. The authors pointed out that the results show supra-threshold spatial additivity across a large region of visual space and that to produce these lateral effects on the target patch the outputs of early visual mechanisms with small receptive fields (retinal ganglion cells and LGN cells) must be summed at some stage in a manner that results in point-by-point additivity. Further studies using radial sinusoidal patterns (Zaidi and Zipser, 1993) strongly supported these findings. As predicted, no contrast induction in the target patch was observed using this pattern since the induction from points at the same distance from the target canceled when summed. Spehar et al. (1996) extended these tests for spatial additivity to brightness induction from complex non-figural achromatic surrounds composed of binary random texture.

Shapley and Reid (1985) and Reid and Shapley (1988) illustrated the effect of distant regions on target patch brightness using stimuli in which equiluminant target regions were embedded in equiluminant annuli (adjacent surrounds) and it was only the outer (remote) backgrounds that differed from each other. Note that, as observed in simple target and surround stimuli like simultaneous brightness contrast, the perceived intensity (brightness) of the equiluminant adjacent surrounds differed in the direction of brightness contrast from their remote backgrounds. The adjacent surround on the high luminance remote background looked darker than the adjacent surround on the low luminance remote background. Of particular interest, however, was that the target region on the high luminance remote background also appeared darker than the target region on the low luminance remote background. This means that the perceived intensity (brightness) of the target cannot be explained by a mechanism that depends solely on either the veridical luminance or the perceived intensity (brightness) of the adjacent surround or border but is also influenced in the direction of contrast from more distant regions.

Reid and Shapley (1988) cautioned that these more complex stimuli are particularly subject to the terminological confound discussed earlier. On a purely descriptive level, the brightness of the target in these stimuli can be described as due to a contrast effect across distance from the remote background or as due to an assimilation effect from the adjacent surround. This emphasizes that the terms brightness contrast and brightness assimilation need to be clearly defined when discussing mechanisms as opposed to simply describing the direction of an effect with respect to an arbitrary reference area or edge. Here we use the term “remote contrast effect” to avoid confusion with descriptions of contrast or assimilation in simple stimuli where assimilation, as discussed earlier, is generally observed only in displays containing high spatial frequency patterns (Helson, 1963; DeValois and DeValois, 1988; Smith et al., 2001; Blakeslee and McCourt, 2004) and results from optical and/or neural blurring.

Reid and Shapley (1988) manipulated both the contrast (+25% to −21%) of the remote backgrounds with the adjacent surround (luminance = 70 cd/m^2^) and the width of the adjacent surround (5’ to 43’ of visual angle or 0.08°, 0.18°, 0.35°, 0.53°, 0.72°). They concluded that under their conditions the remote contrast effect (the effect of the remote background on the 11’ radius target patch) was less than the adjacent contrast effect (the effect of the remote background on the adjacent surround brightness) and that the remote contrast effect decreased as a function of distance between the target edge and the remote background luminance edge (i.e., with the width of the adjacent surround). Reid and Shapley (1988) modified Land and McCann’s early edge-integration (Retinex) model (Land and McCann, 1971) to explain the brightness of the target patch. In Reid and Shapley’s feed-forward edge-integration contrast model (Reid and Shapley, 1988) the contrast information from each edge was combined with the appropriate distance-determined weight to predict target brightness. Although edge based, rather than point-by-point, this feed-forward model agrees with the findings of Zaidi et al. (1992), Zaidi and Zipser (1993), Spehar et al. (1996), and Hong and Shevell (2004) that the effect of distant regions decreases with distance from the target region.

Vladusich et al. (2006) also studied concentric target disk/adjacent surround-ring/remote background stimuli to investigate how signals from local and remote regions interact. Vladusich et al. (2006) examined various edge-integration models using stimuli with opposite and same polarity local and remote induction edges. To produce these edge relationships Vladusich et al. (2006) used a procedure in which they varied the luminance of a 2.0° target (reference) disk (29.9–83.3 cd/m^2^) in a constant 50 cd/m^2^ adjacent surround-ring (2.0°), on remote backgrounds of 10, 40, 60, 90 cd/m^2^. The matching disk was the same size as the target (reference) disk and was presented on the remote background (no surround-ring). These investigators concluded that while same polarity edge signals produced same polarity contrast effects on the target disk, opposite polarity edge signals produced contrast signals that were opposing. They argued that the balance between these opposing signals depended on the relative strength of the induction signals from these edges such that a high-contrast remote induction signal can completely overwhelm the opposing signal from an adjacent surround-ring. Although they referred to this effect as an assimilation effect, in terms of mechanism it is actually the “contrast at a distance” effect (what we refer to as remote contrast in this article) coined by Reid and Shapley (1988). Similar to earlier studies (Reid and Shapley, 1988, Hong and Shevell, 2004), Vladusich et al. (2006) found that the largest effect from an opposing remote background signal occurred in their condition in which the remote background had the lowest luminance (10 cd/m^2^) and that irrespective of the polarity between local and remote inducers, their matching data were best described by a model in which gain varied with background luminance.

Around the same time Rudd and Zemach (2007) also examined concentric target (radius 0.35°) and adjacent surround-ring (width 0.35°) stimuli in a remote background. Recall that Vladusich et al. (2006) varied the target patch and remote background luminance and held the adjacent surround constant. Rudd and Zemach (2007) held the target patch constant and varied the adjacent surround-ring luminance and remote background luminance over a reduced luminance range. Like Vladusich et al. (2006), the goal of Rudd and Zemach’s study (Rudd and Zemach, 2007) was to test various iterations of their own edge integration model by which the remote background/adjacent surround-ring edge and the adjacent surround-ring/target edge combine to predict target patch matching luminance (brightness). Rudd and Zemach (2007) compared matching data from subjects under four stimulus conditions that they designated: decrement-decrement (Dec-Dec); decrement-increment (Dec-Inc); increment-decrement (Inc-Dec); and increment-increment (Inc-Inc). These terms describe the two luminance edges of the stimulus starting from the remote background and moving inward to the target disk. For example, in the Dec-Dec stimuli the remote background luminance was higher (5.89 cd/m^2^) than the adjacent surround-ring luminance (1.48–4.47 cd/m^2^); and therefore, the edge between the remote background and adjacent surround-ring was labeled a decrement. Similarly, the adjacent surround-ring luminance (1.48–4.47 cd/m^2^) was higher than the target-disk luminance (1.0 cd/m^2^) and also labeled a decrement. Note that we follow this naming convention in the present article, however, it is important to be aware that the starting direction is arbitrary. For example, in Vladusich et al. (2006), the opposite-polarity conditions are discussed with the names reversed. Rudd and Zemach (2007) concluded that their results were consistent with an edge-integration model in which the contrast magnitude and polarities of the edges between the adjacent surround-ring and target, and between the remote background and adjacent surround-ring determine whether a remote induction signal originating from the remote background/adjacent surround-ring edge is either partially attenuated (blocked) or amplified by the adjacent surround-ring/target edge. In other words, Rudd and Zemach (2007) included a gain parameter that could be attenuating or amplifying depending on the magnitude and configuration of edges in the stimulus. Specifically, Rudd and Zemach (2007) proposed that the strength of induction from the remote background/adjacent surround-ring edge is attenuated (decreased) by a target disk/adjacent surround-ring border in which the target disk is a decrement compared to the adjacent surround-ring (Dec-Dec and Inc-Dec) and is amplified (increased) by a target disk/adjacent surround-ring border in which the target disk is an increment compared to the adjacent surround-ring (Dec-Inc and Inc-Inc). Rudd and Zemach (2007) note, however, that an equally plausible model is one in which the gain control runs in the opposite direction such that the strength of induction from the target disk/adjacent surround-ring edge is either partially blocked or else amplified by the remote background/adjacent surround-ring border. In this case, the strength of induction from target disk/adjacent surround-ring edge is attenuated (decreased) by a remote background/adjacent surround-ring border in which the adjacent surround-ring is a decrement compared to the remote background (Dec-Dec and Dec-Inc) and is amplified (increased) by a remote background/adjacent surround-ring border in which the adjacent surround-ring is an increment compared to the remote background (Inc-Dec and Inc-Inc). Rudd and Zemach (2007) acknowledged that their results and conclusions are quite different from those of Vladusich et al. (2006), discussed above, where same polarity (Inc-Inc and Dec-Dec) and opposite polarity (Dec-Inc and Inc-Dec) edges produce same and opposite polarity contrast signals that combine to control target patch matching luminance.

The current study resolves some of the above differences regarding the induction effects of adjacent and remote backgrounds on target patches not through modeling (or curve fitting) their combined effects, but by experimentally isolating the effects of both surround-ring luminance and remote background luminance on target patch matching luminance over a wide range of parameters. This approach was inspired by the work of Reid and Shapley (1988), discussed earlier, in which they experimentally isolated the influence of a remote background on target patch matching luminance. To accomplish our experimental goal, we examine the effect of both adjacent surround-ring width and luminance in the presence of three remote background luminances. Specifically, Experiment 1 psychophysically isolated in four observers the effect on target patch matching luminance of six adjacent surround-ring widths (0.1°–24.5°) varied over 11 adjacent surround ring luminances (32–96 cd/m^2^) on a remote background that had the same mean luminance as the target (64 cd/m^2^). Experiment 2 examined, in the same four observers, the effect of these identical conditions on target patch matching luminance in the presence of both a dark (0.0 cd/m^2^) and a bright (96 cd/m^2^) remote background. Finally, we differenced the results of Experiment 1 (the isolated effect of the adjacent surround-ring) from those of Experiment 2 (the combined effect of the adjacent surround-ring with the dark and bright remote backgrounds) to obtain a view of the data that, to at least a first approximation, further isolated the effect of the remote background on target brightness.

Our results support those of Vladusich et al. (2006) as opposed to Rudd and Zemach (2007). The experiments reveal that same-polarity contrast signals (Inc-Inc and Dec-Dec) from adjacent and remote regions produce same-polarity brightness contrast effects on the target patch resulting in the greatest magnitude of brightness (Inc-Inc) and darkness (Dec-Dec) induction respectively. Opposite-polarity contrast signals (Inc-Dec and Dec-Inc) result in a decrease in the overall magnitude of induction and clearly demonstrate that the magnitude and under some conditions the direction of induction (brightening or darkening of the test patch relative to veridical) is controlled by the balance of the induction from the remote background and the adjacent surround.

In addition, we argue based on the literature reviewed above that induction effects are unlikely to be based purely on the “filling-in” of edge signals as implied by edge-integration models. As discussed by Kingdom (2011), all edge-integration models integrate edge signals across space to predict brightness/lightness values. When stimuli are relatively simple this approach is feasible, however, it quickly becomes intractable as scenes increase in complexity. Modeling of these data based on multiscale spatial filtering (for example, the ODOG model) is beyond the scope of this report but will be the topic of a subsequent article.

## Materials and methods

### Observers

Two male (ages 30 and 62) and two female (ages 21 and 62) with normal or corrected-to-normal vision participated in the experiment. Each participant provided informed consent and the experimental protocol was approved by the NDSU Institutional Review Board.

### Stimuli

**Figure 1:**
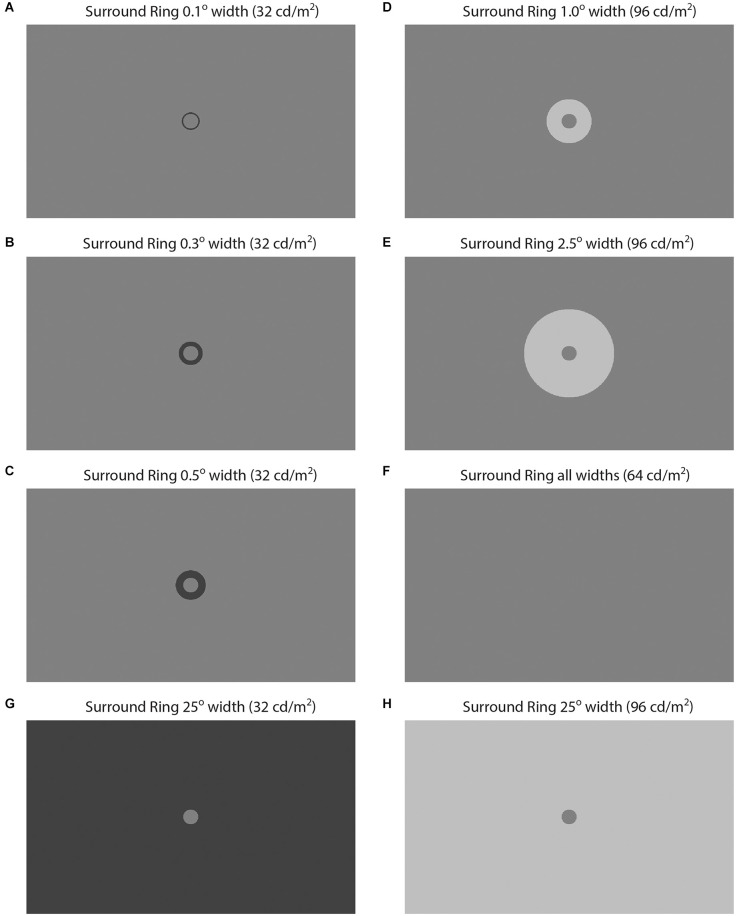
Illustration of some of the brightness induction stimuli from Experiment 1. In **(A–H)** circular target patch luminance is 64 cd/m^2^ and target patch radius is 0.5°. In **(A–F)** the remote background is 64 cd/m^2^ and the adjacent surround-ring varies in luminance and width. In **(A–C)** adjacent surround-ring luminance is 32 cd/m^2^ and adjacent surround-ring width is **(A)** 0.1°, **(B)** 0.3°, and **(C)** 0.5°. In **(D,E)** adjacent surround-ring luminance is 96 cd/m^2^ and adjacent surround-ring width is **(D)** 1.0° and **(E)** 2.5°. In **(F)** adjacent surround-ring luminance is 64 cd/m^2^. Since the target patch and remote background luminance are also 64 cd/m^2^ this stimulus reduces to homogeneous field for all adjacent surround-ring widths. In **(G,H)** adjacent surround-ring width is expanded to 25° such that there is no remote background. In **(G)** the luminance of the adjacent surround-ring is 32 cd/m^2^ and in **(H)** it is 96 cd/m^2^.

Stimuli were presented on a second-generation high-dynamic-range (HDR) monitor (HDR47E, Sim2 Multimedia, S.p.A., Pordenone, Italy) comprised of a 47”, 60 Hz LCD display with 1,920 × 1,080 pixel front-panel resolution, and a backlight matrix consisting of a hexagonal array of 2,202 high intensity LEDs. Stimuli were generated using MATLAB routines and presented as pseudo-grayscale images possessing 1,000 linear intensity steps using the bit-stealing method of (Tyler et al., 1992). Gamma correction was accomplished *via* look-up tables created based on photometric calibration (ColorCal, Cambridge Research Systems, Rochester, Kent, UK). The 105 cm × 59 cm display was viewed at 110 cm and subtended 50° × 29° of visual angle. The sequencing and timing of image presentation and the collection of observer responses was controlled using Presentation (Neurobehavioral Systems, Inc., Berkeley, CA, USA). The target patch, adjacent surround-ring, and remote background of the stimuli occupied the upper two-thirds of the display. The radius of the circular target patch was 0.5° in all conditions. In Experiment 1 the adjacent surround-ring widths were 0.1°, 0.3°, 0.5°, 1.0°, 2.5° or 24.5° such that the target patch plus adjacent surround-ring radii measured 0.6°, 0.8°, 1.0°, 1.5°, 3.0°, and 25°. Target patch luminance was held constant at 64 cd/m^2^. For each of the six adjacent surround-ring sizes, the surround-ring luminance was varied from 32 to 96 cd/m^2^ (32.0, 38.4, 44.8, 51.2, 57.6, 64.0, 70.4, 76.7, 83.1, 89.5, 95.9 cd/m^2^) on a background 64 cd/m^2^. Figure 1 illustrates examples of these stimuli where panels A–E depict the 0.1°, 0.3°, 0.5°, 1.0°, and 2.5° surround-ring widths on the 64 cd/m^2^ remote background, respectively. In panels A–C adjacent surround-ring luminance is 32 cd/m^2^, in panels D and E adjacent surround-ring luminance is 96 cd/m^2^. Note that in panel F surround-ring luminance is 64 cd/m^2^. For all surround-ring sizes this surround-ring luminance results in the stimulus reducing to a homogeneous field since target patch, adjacent surround-ring, and remote background are all 64 cd/m^2^. Finally, panels G and H illustrate the expanded adjacent surround-ring condition (24.5° width). This expanded surround-ring covered the full extent of the stimulus display (50° × 29°) resulting in a target patch (0.5° radius) in a large adjacent surround. As in the other conditions, this large adjacent surround varied over the full range of adjacent surround-ring luminance. Panels G and H illustrate this stimulus when adjacent surround-ring luminance was at the extremes of this range (i.e., 32 cd/m^2^ and 96 cd/m^2^).

**Figure 2:**
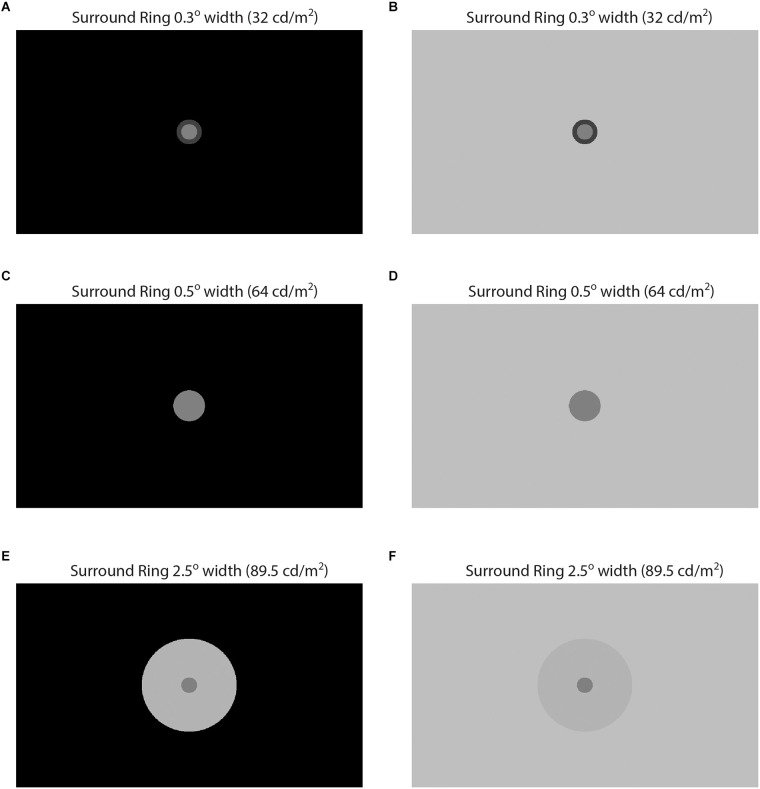
Illustration of some of the brightness induction stimuli from Experiment 2. In **(A–F)** target patch luminance is 64 cd/m^2^ and target patch radius is 0.5°. In **(A,C,E)** the remote background is 0.0 cd/m^2^ and in **(B,D,F)** the remote background is 96 cd/m^2^. The adjacent surround-ring varies in luminance and width but is identical for pairs **(A,B)**, **(C,D)**, and **(E,F)**. In pair **(A,C)** the adjacent surround-ring is 0.3° in width and its luminance is 32 cd/m^2^. In pair **(C,D)** the adjacent surround-ring is 0.5° in width and its luminance is 64 cd/m^2^. Since the target patch and the adjacent surround-ring have the same luminance in this pair they are not distinguishable, and the stimulus reduces to a circular patch with a radius of 1°. In pair **(E,F)** the adjacent surround-ring is 2.5° in width and its luminance is 89.5 cd/m^2^.

Experiment 2 involved the same observers and was similar to Experiment 1 in that adjacent surround-ring width and luminance were again varied. In Experiment 2, however, the remote background luminance was either 0.0 or 96 cd/m^2^ (rather than 64 cd/m^2^) and the large adjacent surround was not retested. Note that 0.0 cd/m^2^ refers to a photometric reading of less than 0.005 cd/m^2^ on the high dynamic range display. Figure 2 illustrates examples of targets with three adjacent surround-ring widths (0.3°, 0.5°, and 2.5°) at three luminance levels (32 cd/m^2^, 64 cd/m^2^, and 89.5 cd/m^2^) on each of the two remote backgrounds (0.0 and 96 cd/m^2^). The upper pair of panels (A,B) show identical target patch (0.5° radius, 64 cd/m^2^) and adjacent surround-ring (0.3° width, 32 cd/m^2^) stimuli on the 0.0 cd/m^2^ and 96 cd/m^2^ remote backgrounds, respectively. Likewise, the bottom pair of panels (E,F) depict identical target patch (0.5° radius, 64 cd/m^2^) and adjacent surround-ring (2.5° width, 89.5 cd/m^2^) stimuli on the same two remote backgrounds (0.0 and 96 cd/m^2^). Note that in the middle pair of panels (C,D) adjacent surround-ring luminance is equal to the target patch luminance (64 cd/m^2^) resulting in a stimulus discontinuity that reduces to a simple target patch in a large contiguous surround. These stimuli appear similar to the large surround-ring stimuli from Experiment 1 (see Figures 1G,H); however, the target patch in Figures 2C,D is larger since the target patch size now equals the size of the target patch plus adjacent surround-ring (in this instance 1.0° radius) as opposed to the radius of the target patch alone (0.5°).

In both Experiments 1 and 2 square matching patches (1° × 1°) were presented on a checkerboard background (contrast = 0.30, check luminance = 54.4 cd/m^2^ and 73.6 cd/m^2^) that was vertically centered in a homogeneous field (64 cd/m^2^) which occupied the lower third of the display. The checkerboard background measured 2.0° H × 2.0° W and the individual checks measured 0.25° × 0.25°.

### Procedures

A method of adjustment matching procedure was employed to measure induction magnitude. Observers adjusted matching patch luminance using a mouse wheel. Each click of the mouse wheel adjusted the luminance of the matching patch by 0.1%. Matching patch luminance for each target patch was obtained as a function of adjacent surround-ring size and luminance on the 64 cd/m^2^ remote background (Experiment 1) and on the 0.0 cd/m^2^ and 96 cd/m^2^ remote backgrounds (Experiment 2). Observers were instructed to adjust the luminance of the matching patch such that its perceived intensity (brightness) matched that of the target patch. Note that because illumination is unambiguously homogeneous in these displays (i.e., there are no illumination boundaries such as shadows present in the stimuli) instructions to match perceived intensity (brightness) produce the same matching luminances as instructions to match perceived reflectance (lightness) (Arend and Spehar, 1993; Blakeslee and McCourt, 2012, 2015a,b). A checkerboard background for the matching patch was employed to ensure that brightness-contrast (i.e., the intensity ratio at the border between the matching patch and its background) was not the dimension of visual experience being matched (Arend and Spehar, 1993; Blakeslee and McCourt, 2012, 2015a,b). Previous investigations noted that observers report brightness matching to be easier on a checkerboard background although no differences in matching results were observed between homogeneous and checkerboard backgrounds with the same mean luminance (Blakeslee et al., 2005). In Experiments 1 and 2 the various combinations of adjacent surround-ring size and luminance were presented randomly within a block of trials. In Experiment 2 the different remote backgrounds were presented such that all stimuli appeared first on the 0.0 cd/m^2^ remote background followed by the 96 cd/m^2^ remote background or *vice versa*. A complete block of matching trials in Experiment 1, one match per condition, consisted of 66 matches and each observer completed 10 blocks. In Experiment 2 a complete block of trials consisted of 110 matches and each observer again completed 10 blocks.

## Results

**Figure 3:**
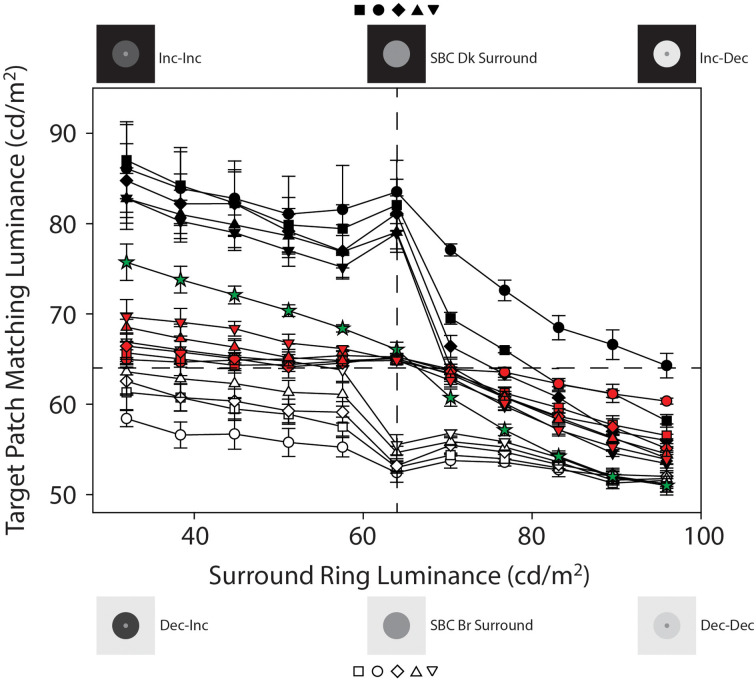
Mean target patch matching luminance from Experiments 1 and 2 plotted as a function of adjacent surround-ring luminance, averaged across the four observers (±1 SEM). The symbol shapes represent the various adjacent surround-ring widths (0.1° = circles, 0.3° = squares, 0.5° = diamonds, 1.0° = triangles, 2.5° = inverted triangles). The red, black, and white symbol colors indicate the three different remote background luminance conditions on which the target patch plus adjacent surround-ring combinations were presented (red = 64 cd/m^2^, black = 0.0 cd/m^2^, white = 96 cd/m^2^). The green stars plot the results for the condition in which the adjacent surround-ring filled the entire background of the stimulus (50°). The surround-ring in this condition assumed the same luminance values (32 cd/m^2^–96 cd/m^2^) as the adjacent surround-rings in the other conditions. The horizontal dashed line represents a veridical luminance match to the target patch (64 cd/m^2^) and the vertical dashed line indicates the location where adjacent surround-ring luminance equals target patch luminance. The stimulus icons appearing at the top and bottom of Figure 3 depict the luminance relationships between the target patch (64 cd/m^2^) and variable adjacent-surround rings, on the dark and bright remote backgrounds (see text for details).

**Figure 4:**
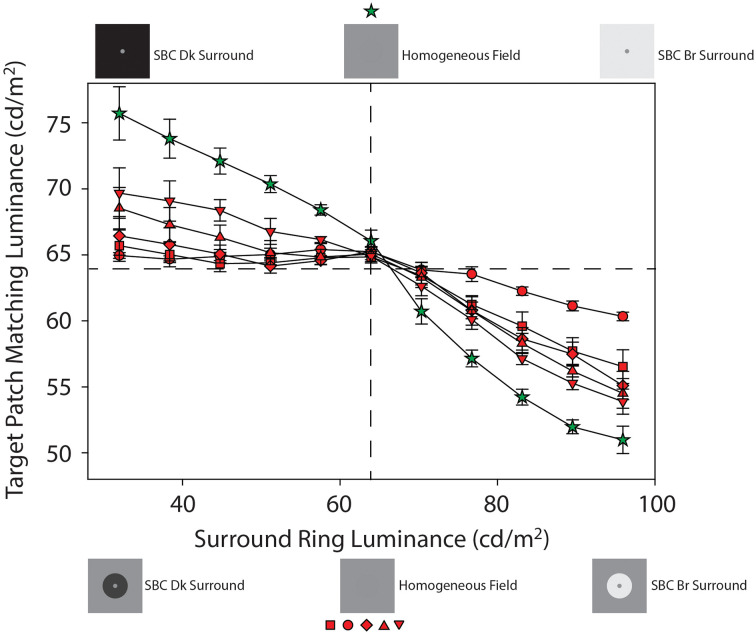
Mean target patch matching luminance from Experiment 1 plotted as a function of adjacent surround-ring luminance on the 64 cd/m^2^ remote background (red symbols) and on the full-field adjacent surround (green stars). These data from Figure 3 are plotted on an expanded target patch matching luminance axis for clarity. The red symbol shapes represent the various adjacent surround-ring widths (0.1° = circles, 0.3° = squares, 0.5° = diamonds, 1.0° = triangles, 2.5° = inverted triangles). Adjacent surround-ring luminance in each condition ranged from 32 cd/m^2^ to 96 cd/m^2^. The stimulus icons at the top of the figure depict examples of the stimuli in the extended (full field) surround-ring matching condition represented by the green stars. The stimulus icons at the bottom of figure depict examples of the stimuli for the smaller adjacent surround-ring widths (red symbols) on the 64 cd/m^2^ remote background (see text for details).

Figure 3 plots mean target patch matching luminance from Experiments 1 and 2, averaged across four observers (+ or −1 SEM), as a function of adjacent surround-ring luminance. The symbol shapes represent the various adjacent surround-ring widths (0.1° = circles, 0.3° = squares, 0.5° = diamonds, 1.0° = triangles, 2.5° = inverted triangles). The red, black, and white symbol colors indicate the three different remote background luminance conditions on which the target patch plus adjacent surround-ring combinations were presented (red = 64 cd/m^2^, black = 0.0 cd/m^2^, white = 96 cd/m^2^). The green stars are the results for the expanded adjacent surround-ring condition in which the adjacent surround-ring filled the entire background of the stimulus. The horizontal dashed line represents a veridical luminance match to the target patch (64 cd/m^2^) and the vertical dashed line indicates the location where adjacent surround-ring luminance equals target patch luminance. The stimulus icons appearing at the top and bottom of Figure 3 depict the luminance relationships between the target patch (64 cd/m^2^) and variable adjacent-surround rings, on the dark (see stimulus Figures 2A,E) and bright (see stimulus Figures 2B,F) remote backgrounds. For clarity, the matching functions (and associated stimulus icons) for the variable adjacent surround-rings on the 64 cd/m^2^ background (red symbols) and for the full field variable adjacent-surrounds (green stars) are replotted in Figure 4 with an expanded target patch matching luminance axis and are discussed later. In Figure 3 note that when adjacent surround-ring luminance is less than target patch luminance (64 cd/m^2^, vertical dashed line) the stimulus icons for the dark (top left) and bright (bottom left) remote backgrounds are labeled Inc-Inc and Dec-Inc, respectively, following the naming convention of Rudd and Zemach (2007). Recall that according to this convention the first term describes the transition between the dark (0.0 cd/m^2^) or bright (96 cd/m^2^) remote background and the variable luminance (32.0–57.6 cd/m^2^) adjacent surround-ring. On the dark remote background this transition is an increment (Inc) and on the bright remote background it is a decrement (Dec). The second term describes the luminance transition between the variable (32.0–57.6 cd/m^2^) adjacent surround-ring and the target patch (64 cd/m^2^). These values are the same irrespective of the dark or bright remote background and the second term is an Inc for both. When adjacent surround-ring luminance is greater than target patch luminance (64 cd/m^2^), the stimulus icons on the dark (top right) and bright (bottom right) remote backgrounds are labeled Inc-Dec and Dec-Dec, respectively. The middle icons depict the stimulus on the dark (upper) and bright (lower) remote background when adjacent surround-ring luminance is the same as target luminance (64 cd/m^2^). At this location the stimulus reduces to a target patch of larger size (equal to the size of the target patch plus adjacent surround-ring) on the dark or bright remote background (Figures 2C,D).

Recall that the green stars (Figures 3 and 4) show the effect of the adjacent surround-ring when it fills the entire background (50°) and that the red symbols represent the various smaller adjacent surround-ring widths on a remote background that is the same luminance as the target patch (64 cd/m^2^). Since the remote background of 64 cd/m^2^ is not expected to induce a brightness change in a target patch of the same luminance, the green and red matching functions represent the isolated effect of adjacent surround-ring luminance (32–96 cd/m^2^) on target patch matching luminance at each adjacent surround-ring size. The stimulus icons at the top of 4 depict examples of the stimuli in the extended (full field) surround-ring matching condition represented by the green stars (see stimulus Figures 1G,H). The stimulus icons at the bottom of Figure 4 are examples of the stimuli for the smaller adjacent surround-ring widths (red symbols) on the 64 cd/m^2^ remote background (see stimulus Figures 1A–E). As in Figure 3, the icons on the left side (top and bottom) depict the luminance relationship between the target patch (64 cd/m^2^) and the adjacent surround-rings when surround-ring luminance is below 64 cd/m^2^. The icons on the right side (top and bottom) depict the luminance relationship between the target patch (64 cd/m^2^) and the adjacent surround rings when surround ring luminance is above 64 cd/m^2^. The middle icons depict the stimulus when the adjacent surround-rings are the same luminance as the target patch (64 cd/m^2^). For all adjacent surround-ring sizes the stimulus at this point becomes a 64 cd/m^2^ homogeneous field (see stimulus Figure 1F).

From the simultaneous contrast literature, we know that induction in a target patch increases in magnitude with increasing surround size (Wallach, 1948; Diamond, 1953, 1955; Stevens, 1967; Heinemann, 1972; Yund and Armington, 1975). In agreement with this literature, the condition in which the adjacent surround-ring fills the entire field (Figures 3 and 4, green stars) produces the maximum brightness induction (highest target patch matching luminance) at the lowest adjacent surround-ring luminance (32 cd/m^2^). Matching luminance declines steadily as adjacent surround-ring luminance increases, passes close to veridical matching when surround-ring luminance equals the luminance of the target (i.e., the stimulus is a 64 cd/m^2^ homogeneous field), and continues to decline reaching the point of maximum darkness induction (lowest target patch matching luminance) when adjacent surround-ring luminance is highest (96 cd/m^2^). Note that the matching function is asymmetric in that adjacent surround-ring luminances that are less than target patch luminance (64 cd/m^2^) have less of an induction effect on the target (brightening) than adjacent surround-rings that are greater in luminance than the target patch (darkening). This asymmetry is also seen for the smaller width adjacent surround-rings on the 64 cd/m^2^ background (Figures 3 and 4, red symbols). The asymmetry agrees with previous reports that surrounds that are lower in luminance than the target patch have less impact on target patch brightness compared to surrounds that are higher in luminance than the target patch (Diamond, 1953, 1955; Heinemann, 1972). Plotting the functions on logarithmic axes, as in these previous studies, emphasizes this asymmetry.

In addition, as expected, the overall magnitude of induction shows an orderly decline with decreasing adjacent surround-ring size. Again, at an adjacent surround-ring luminance of 64 cd/m^2^ (dashed vertical line) the target patch, adjacent surround-ring, and remote background are all equal in luminance (64 cd/m^2^) resulting in the reduction of the stimulus to a homogeneous field for all adjacent surround-ring sizes (Figure 1F). In accord with this, the matching functions represented by both the red and green symbols all converge at this location near veridical matching (64 cd/m^2^, horizontal dashed line).

The filled (black) symbols (Figure 3) depict target patch matching luminance as a function of adjacent surround-ring luminance for different surround-ring widths on the dark (0.0 cd/m^2^) remote background (see stimulus Figures 2A,C,E). First, note in Figure 3 that the upward shift (increase in target patch matching luminance) of the matching functions (black symbols), compared to the functions on the 64 cd/m^2^ remote background (red symbols), represents the brightening effect of the dark (0.0 cd/m^2^) remote background on the matching functions. The magnitude of this effect of the remote background depends on both adjacent surround-ring luminance and width. Target patch matching luminance is greatest on the dark (0.0 cd/m^2^) remote background when adjacent surround-ring luminance is less than target patch luminance (64 cd/m^2^). In this region (Inc-Inc), the remote background (0.0 cd/m^2^) and the adjacent surround-ring (34–58 cd/m^2^) are both darker than the target patch (64 cd/m^2^). Comparing these functions with those on the 64 cd/m^2^ background (red symbols) indicates that the induction effect on the target patch from the remote background and the adjacent surround-ring are both in the direction of contrast (brightening). The overall upward shift is greatest when the luminance of the adjacent surround-ring is lowest and declines as adjacent surround-ring luminance increases.

Second, brightening from the remote background is moderated by adjacent surround-ring width; induction (brightening) is greatest for the target patches with the smallest adjacent surround-ring widths (black circles and squares) and least for the target patches with the largest adjacent surround-ring width (black inverted triangles). Note that this order is reversed from what is observed on the 64 cd/m^2^ remote background (red symbols) because the dark remote background has more of an induction effect on the target patches with the smaller adjacent surround-ring widths.

Third, the decline in target patch matching luminance as adjacent surround-ring luminance increases from 32 to 58 cd/m^2^ reverses temporarily at an adjacent surround-ring luminance of 64 cd/m^2^. This reversal is explained by the stimulus discontinuity at this location mentioned previously. When adjacent surround-ring luminance and target patch luminance are both 64 cd/m^2^ they are not distinguishable and the stimulus reduces to a target patch of larger size, equivalent to that of the target patch plus the adjacent surround-ring, in a dark background. Since the stimuli now correspond to the classic simultaneous contrast configuration, in accord with this literature (Wallach, 1948; Diamond, 1962; Stevens, 1967; Heinemann, 1972; Yund and Armington, 1975), the stimulus with the smallest target patch size (black circles) shows the greatest overall magnitude of brightness induction from the dark background and the stimulus with the largest target patch (black inverted triangles) shows the smallest overall magnitude of induction.

Fourth, the reappearance of the adjacent surround-ring when the surround-ring luminance exceeds 64 cd/m^2^ results in a sudden and relatively steep decline in target patch matching luminance. In this region (Inc-Dec) the induction effect (brightening) from the dark (0.0 cd/m^2^) remote background is strongly counteracted by the induction effect (darkening) due to the increasing luminance of the adjacent surround-ring. As shown by Heinemann (1955), darkness induction from an adjacent surround-ring is quite pronounced under these conditions.

**Figure 5:**
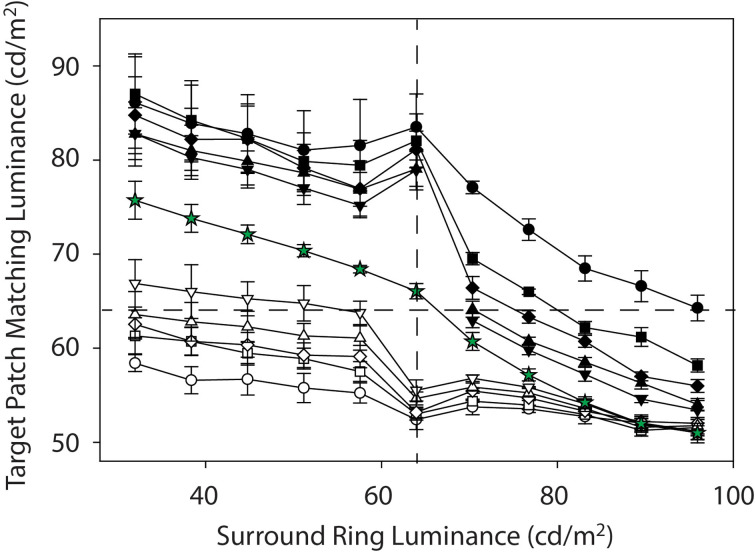
Mean target patch matching luminance on the 0.0 cd/m^2^ remote background (black symbols) and on the 96 cd/m^2^ (white symbols). Symbol shapes represent the various adjacent surround ring widths (0.1° = circles, 0.3° = squares, 0.5° = diamonds, 1.0° = triangles, 2.5° = inverted triangles). The matches obtained on the variable full-field surround (green stars) are included for reference while those on the 64 cd/m^2^ background (red symbols) have been removed for clarity.

Again, the counteracting darkness induction from the adjacent surround-ring is greatest in overall magnitude for the target patches with adjacent surround-rings of the largest width (black inverted-triangles) and least for target patches with adjacent surround-rings of the smallest widths (black circles). This can be clearly seen in Figure 5 where the target patch matching functions on the 64 cd/m^2^ background (red symbols) have been removed. Note that for all but the smallest width adjacent surround-ring (black circles) the functions on the 0.0 cd/m^2^ remote background (black symbols) eventually drop to target matching luminances below veridical and come close to those on the 64 cd/m^2^ (red symbols) remote background (Figure 3). Indeed, induction magnitude for the largest adjacent surround-ring on the 0.0 cd/m^2^ remote background (black inverted triangles) reaches the point of maximum darkness induction in this study (green stars) at an adjacent surround-ring luminance of 96 cd/m^2^ (Figure 5). Recall that Vladusich et al. (2006) found that observers matched local increments to decrements and *vice versa* when the remote edge was of opposite polarity and high contrast relative to the local edge. Matching a local decrement with an increment, is apparent in the current Inc-Dec region (filled black symbols for adjacent surround-ring luminances greater than 64 cd/m^2^) for all points above the horizontal dashed line (veridical target patch matching luminance). Only the mean target patch matching luminance for the target patch with the narrowest width adjacent surround-ring (black circles), however, remains above veridical at the highest surround-ring luminance (96 cd/m^2^). This makes sense since, as previously mentioned, the remote background has greater influence (and the adjacent surround-ring less influence) on target patches with narrower surrounds. In this Inc-Dec region the balance between the remote background and the adjacent surround-ring appears to favor the adjacent surround.

The open (white) symbols (Figures 3 and 5) depict mean target patch matching luminance as a function of adjacent surround-ring luminance for different surround-ring widths on the bright (96 cd/m^2^) remote background (see stimulus Figures 2B,D,F). First, the overall downward shift of the matching functions (white symbols) compared to the functions on the 64 cd/m^2^ background (red symbols) represents the remote induction (darkening) effect of the bright (96 cd/m^2^) remote background on the matching functions. The magnitude of the induction (darkening) effect from the remote background again depends on both adjacent surround-ring luminance and width. Darkening from the bright (96 cd/m^2^) remote background is least when adjacent surround-ring luminance is less than target patch luminance (64 cd/m^2^). This makes sense because in this stimulus region (Dec-Inc) the inducing influence of the bright remote background (darkening) and the dark adjacent surround-ring (brightening) are in opposition. Here, however, the magnitude and rate of decline is much less than occurred for the opposing stimulus condition on the dark remote background (Inc-Dec, filled symbols for adjacent surround-rings greater than 64 cd/m^2^).

Second, the magnitude of the induction (darkening) from the bright remote background also depends on adjacent surround-ring width. When the remote background (96 cd/m^2^) is brighter than the adjacent surround-ring (Dec-Inc), darkness induction from the 96 cd/m^2^ remote background is greatest for the target patches with the smallest adjacent surround-ring width (open circles) and least for the target patches with the largest adjacent surround-ring width (open inverted triangles). Note that these data are again consistent with the findings of Vladusich et al. (2006) where observers matched local increments to decrements and* vice versa* when the remote edge was of opposite polarity and high contrast relative to the local edge. In the Dec-Inc region this behavior (matching a local increment with a decrement) is apparent for all points below the horizontal dashed line (veridical target patch matching luminance). Here only the mean target patch matching luminance for the target patch with the largest adjacent surround-ring width (open inverted triangles) remains above veridical matching. This makes sense since induction from the remote background is least for the target patches with the largest adjacent surround-ring widths. Note that in this Dec-Inc region (as opposed to the Inc-Dec region discussed earlier) the balance between the remote background and the adjacent surround favors the remote background.

Third, as the adjacent surround-ring luminance approaches 64 cd/m^2^, the modest decline in target patch matching luminance as adjacent surround-ring luminance increases from 32 to 57.6 cd/m^2^ is accelerated (rather than reversed as seen on the dark remote background). This accelerated decline is again explained by the stimulus discontinuity at this location. When adjacent surround-ring luminance and target patch luminance are both 64 cd/m^2^ they are not distinguishable and the stimulus reduces to a simple simultaneous contrast stimulus (i.e., a target patch of larger size equivalent to the size of the target patch plus adjacent surround-ring, in a bright background). The removal of the counteracting inducing effect (brightening) from the adjacent surround-ring luminance below the target luminance (64 cd/m^2^), results in the appearance of an accelerated decline (darkening) in the target matching luminance due to the bright background. This decline is predictably steepest for the target patches with the largest contiguous surround-rings (open inverted triangles) as the larger surround-rings in the Dec-Inc region exerted more counteracting brightness induction on the target. Since the stimuli now correspond to classic simultaneous contrast stimuli, again in accord with this literature (Wallach, 1948; Diamond, 1955, 1962; Stevens, 1967; Heinemann, 1972; Yund and Armington, 1975), the stimulus with the smallest target patch (open circles) shows the greatest overall magnitude of darkness induction from the background and the stimulus with the largest target patch (open inverted triangles) shows the smallest relative magnitude of induction.

Fourth, as adjacent surround-ring luminance increases above 64 cd/m^2^ (Dec-Dec condition) its effect on the target patch is in the same direction (darkening) as the influence of the remote background. The initial apparent upward inflexion for all adjacent surround-ring sizes is consistent with a recovery from the stimulus discontinuity at 64 cd/m^2^. Note that the overall magnitude of the darkening from both the remote background and adjacent surround-ring in this region (Dec-Dec) is somewhat less than the magnitude of the combined brightening effect in the Inc-Inc condition (black symbols where surround-ring luminance is below 64 cd/m^2^). As expected, however, the remote background again has the strongest influence on the target patches with the narrower adjacent surround-ring widths. Interestingly, as adjacent surround-ring luminance increases in this region, the different adjacent surround-ring size functions appear to compress toward the point of maximum darkness induction in this study. All of the adjacent surround-ring target patch matching functions on the 96 cd/m^2^ remote background (white symbols) predictably converge at an adjacent surround-ring luminance of 96 c/m^2^ since at this point the stimuli all reduce to a 0.5° radius target patch in a full-field 96 cd/m^2^ surround. The functions also overlap at this location with the full field (50°) surround-ring condition (green stars) since this stimulus is also identical at this point and represents the point of greatest darkness induction (Figure 1H).

**Figure 6:**
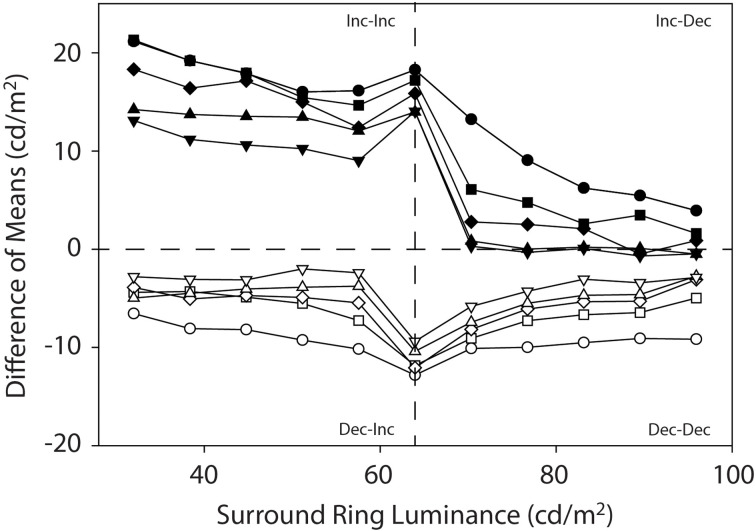
Mean target patch matching luminance plotted as difference functions. Here the filled symbols reflect the difference between the target patch matching functions on the 0.0 cd/m^2^ and 64 cd/m^2^ remote backgrounds and the open symbols plot the difference between the target patch matching functions on the 64 cd/m^2^ and the 96 cd/m^2^ remote backgrounds. This differencing operation is another way to visualize the direction and strength of induction from the dark (0.0 cd/m^2^) and bright (96 cd/m^2^) remote backgrounds in greater isolation from the induction due to the variable adjacent surround-ring luminance (64 cd/m^2^ background). The labels (Inc-Inc, Inc-Dec, Dec-Inc and Dec-Dec) describe the luminance relationships between the remote background, adjacent surround-ring and target patch (see text for details).

Figure 6 illustrates the data from the current experiments as difference functions. Here the filled symbols reflect the difference between the target patch matching functions on the 0.0 cd/m^2^ and 64 cd/m^2^ remote backgrounds and the open symbols plot the difference between the 64 cd/m^2^ and the 96 cd/m^2^ remote backgrounds. This differencing operation is an attempt to visualize the direction and strength of induction from the dark remote (0.0 cd/m^2^) and bright remote (96 cd/m^2^) backgrounds in greater isolation from the induction due to the isolated adjacent surround-ring luminance (red symbols in Figures 3 and 4). Note that if induction from the fixed luminance remote background has a strictly linear effect on target patch brightness, we would expect the difference functions for each surround-ring width to be relatively flat except in the vicinity of the stimulus discontinuity where adjacent surround-ring luminance is 64 cd/m^2^. This is approximately the case for the difference between the target patch matching functions on the 64 cd/m^2^ and 96 cd/m^2^ remote backgrounds (white symbols). The difference functions are all below zero (negative) and appear relatively flat across both the Dec-Inc stimulus region (Figure 6, lower left quadrant) and the Dec-Dec region (Figure 6, lower right quadrant). The difference between the target patch matching functions on the 0.0 cd/m^2^ remote background and the 64 cd/m^2^ remote background (black symbols) are all above zero (positive), however, although some flattening of the functions occurs within the Inc-Inc (Figure 6, upper left quadrant) and Inc-Dec (Figure 6, upper right quadrant) regions, there is a marked difference in the magnitude of the difference between the two regions despite the fixed luminance of the remote background. This behavior indicates that the dark remote background interacts non-linearly with adjacent surround-ring luminance between Inc-Inc and Inc-Dec regions.

The difference functions also add more clarity to the observation that the influence of the remote background on target patch matching depends in an orderly fashion on the surround-ring width. Brightness induction from the 0.0 cd/m^2^ remote background (black symbols) and darkness induction from the 96 cd/m^2^ remote background (white symbols) is greatest for target patches with the smallest width adjacent surround-rings and declines in an orderly fashion as adjacent surround-ring width increases.

## Discussion

The results of the current study resolve differences in the literature regarding the mechanisms responsible for brightness induction in a target patch from adjacent and remote backgrounds by experimentally isolating the effects of both adjacent surround-ring luminance and width, and remote background luminance on target patch matching luminance over a wide range of parameters. To accomplish this, we examined the effect of both adjacent surround-ring width and luminance in the presence of three remote background luminances. Specifically, Experiment 1 psychophysically isolated in four observers the effect on target patch matching luminance of six adjacent surround-ring widths (0.1°–24.5°) varied over 11 adjacent surround ring luminances (32–96 cd/m^2^) on a remote background (64 cd/m^2^) that had the same mean luminance as the target patch (64 cd/m^2^). In other words, Experiment 1 was a classic simultaneous brightness contrast study (red symbols, Figures 3 and 4). Experiment 2 examined, in the same four observers, the effect of the identical adjacent surround-ring luminance and width manipulations, on target patch matching luminance in the presence of a 0.0 cd/m^2^ (Figures 3 and 5, black symbols) and a 96 cd/m^2^ (Figures 3 and 5, white symbols) remote background. These data reveal that same-polarity luminance contrast from adjacent and remote regions (Inc-Inc and Dec-Dec) produces same-polarity brightness induction effects in the target disk, and that opposite-polarity luminance contrast (Inc-Dec and Dec-Inc) produces brightness induction effects that are opposing. Differencing the isolated induction effect of the adjacent surround-ring on target patch brightness (Experiment 1) from the combined induction effect of the adjacent surround-ring with the dark, and separately with the bright remote background from Experiment 2, provides a view of the data that further isolates the effect of the remote background on target patch matching luminance. This alternate view (Figure 6) demonstrates that brightness induction (darkening) in the target from the isolated bright remote background (96 cd/m^2^) is relatively constant in magnitude across all adjacent surround-ring luminances and increases in magnitude with decreasing surround-ring width. Induction (brightening) from the isolated dark remote background (0.0 cd/m^2^) also increased in magnitude with decreasing adjacent surround-ring width. Rather than the dark remote background exerting a constant induction effect (brightening) across all adjacent surround-ring luminances, however, the induction effect from the isolated dark remote background was much reduced in the presence of an adjacent surround-ring of greater luminance than the target patch (Inc-Dec condition) indicating a non-linear interaction between the dark remote background and adjacent surround-ring luminance not observed for the bright remote background.

The results of these experiments support those of Vladusich et al. (2006) as opposed to those of Rudd and Zemach (2007) both of which were discussed earlier. Recall that Vladusich et al. (2006) found that same polarity edge signals produced same polarity contrast effects on the target disk while opposite polarity edge signals produced contrast signals that were opposing. They argued that the balance between these opposing signals depended on the relative strength of the induction signals from these edges such that a high-contrast remote induction signal could completely overwhelm the opposing signal from an adjacent surround-ring. These findings were confirmed by our results. Although they referred to this effect descriptively as an assimilation effect, our data make it clear that in terms of mechanisms it is the “contrast at a distance” effect (what we refer to as remote contrast) coined by Reid and Shapley (1988). Note that Vladusich et al. (2006) as well as Rudd and Zemach (2007) interpreted their results within the framework of edge-integration models. We argue based on the literature reviewed in the “Introduction” Section that these brightness induction effects are unlikely to be based purely on the “filling-in” of edge signals as implied by edge integration models. Modeling of these data based on multiscale spatial filtering is beyond the scope of this report but will be the topic of a subsequent article.

A more recent study by Rudd (2010), using Dec-Dec conditions with two observers, is of interest regarding this region of our data. Rudd (2010) employed target and matching disks of radius 0.35° (similar to the 0.5° radius target and matching disks employed in the current study) with adjacent surround-ring widths of 0.120°, 0.538°, and 1.05° (similar to the 0.1° = circles, 0.5° = diamonds, and 1.0° = triangles, of the current study). According to Rudd (2010), in the presence of a 25.12 cd/m^2^ remote background, increasing the adjacent surround-ring luminance from 1.58 cd/m^2^ to approximately 5 cd/m^2^ increased target disk matching luminance for the two smaller adjacent surround-ring widths at low adjacent surround-ring luminance (which he referred to as assimilation) and decreased the target disk matching luminance at high adjacent surround-ring luminance between 5 and 15 cd/m^2^ (which he described as contrast). In other words, Rudd (2010) reported that assimilation occurred with narrower contiguous surround-rings at low adjacent surround-ring luminance levels, consistent with early studies of assimilation using simple stimuli without a far surround (Jameson and Hurvich, 1961; Helson, 1963).

As discussed in the “Introduction” Section, the descriptive term brightness assimilation refers to a situation when the brightness of the target region shifts toward, rather than away, from that of an adjacent region. It has often been observed in patterns containing high spatial frequencies and is thought to result from a mechanism of optical and/or neural blurring (Helson, 1963; White, 1979; DeValois and DeValois, 1988; Jameson and Hurvich, 1989; Smith et al., 2001; Blakeslee and McCourt, 2004; Hong and Shevell, 2004; Rudd, 2010). Interestingly, Helson (1963) suggested that both area and luminance might be involved in controlling whether assimilation or contrast was observed in his simple stimuli. He argued that larger area and higher luminance acted similarly to push induction from assimilation to contrast. This idea appears to be supported by the Dec-Dec conditions in the Rudd (2010) study which was conducted at much lower luminance levels than the current study. Although we also observe a small inflexion (brightness increase) in the mean data in the Dec-Dec region (Figures 3 and 5, white symbols), unlike Rudd (2010) we see this brightening for all adjacent surround-ring widths. We attribute the brightening in this region of our data to a recovery from the stimulus discontinuity that occurs at an adjacent-surround ring luminance of 64 cd/m^2^ rather than to a mechanism of assimilation (due to optical or neural blurring). Additional support for this interpretation is provided by the finding that we see no evidence of an inflection (brightening) in this region in our 64 cd/m^2^ remote background condition for any contiguous surround-ring size (see Figure 4, red symbols). The matching data in this region for the narrowest surround ring width (0.1°; where assimilation due to optical or neural blurring might be expected) decline slightly with increasing adjacent surround-ring luminance indicating no assimilation (brightening) effect from the low contrast but brighter adjacent surround-ring.

Interestingly, Shevell et al. (1992) approached the problem of induction from adjacent and remote backgrounds in terms of the level in the visual system where these backgrounds exert their effects. Using direct viewing (stimulus presented to both eyes) and haploscopic viewing [stimulus divided by a mirror stereoscope such that the left (right) half of stimulus was directed to only the left (right) eye of the observer], they were able to clearly show that brightness induction from an adjacent surround-ring is dependent on neural mechanisms that precede binocular combination in the cortex. In other words, only the luminance relationship between the target (20’ diameter) and adjacent surround (3° diameter) was important in the determination of target brightness under both conditions. A recent study by Sinha et al. (2020) came to a similar conclusion. Shevell et al. (1992) also found that the influence of the remote background (5° diameter) depended on the binocular fused appearance of the stimulus and therefore was mediated at a cortical level beyond the point of binocular combination. In concert, physiological studies (Reid and Shapley, 1989; Rossi et al., 1996; Rossi and Paradiso, 1999) have demonstrated that brightness processing is not restricted to the retina or LGN but involves the cortex where much larger receptive fields are observed. In cat, for example, Reid and Shapley (1989) were able to show that the responses of retinal ganglion and LGN cells depend on local target-edge contrast while many cortical area-17 cells show influences on target responses from remote backgrounds. Based on these results the brightness contrast induction observed in the target from the adjacent surrounds is likely due to neural mechanisms in the visual system prior to binocular combination in the cortex. Similarly, the brightness contrast induction from the remote backgrounds may originate from neural mechanisms operating both prior to and after binocular combination in the cortex. The differencing operation in Figure 6, our effort to isolate the influence of mechanisms that Shevell et al. (1992) demonstrated are located after binocular fusion in the cortex, indicate that although this mechanism appears to be linear for bright remote backgrounds it is not for dark remote backgrounds.

## Data availability statement

The raw data supporting the conclusions of this article will be made available by the authors, without undue reservation.

## Ethics statement

The studies involving human participants were reviewed and approved by Institutional Review Board (IRB) North Dakota State University. The patients/participants provided their written informed consent to participate in this study.

## Author contributions

All authors contributed to the article and approved the submitted version.
